# Diverse strategies are needed to support physical activity engagement in women who have had breast cancer

**DOI:** 10.1007/s00520-023-08113-7

**Published:** 2023-10-21

**Authors:** Farha Inam, Rebecca J. Bergin, David Mizrahi, David W. Dunstan, Melissa Moore, Natalie Maxwell-Davis, Linda Denehy, Brigid M. Lynch, Christopher T. V. Swain

**Affiliations:** 1 Cancer Science Unit, Faculty of Medicine, Dentistry and Health Sciences, The University of Melbourne, Parkville, Australia; 2 Cancer Epidemiology Division, Cancer Council Victoria, Melbourne, Australia; 3 The Daffodil Centre, The University of Sydney, a joint venture with Cancer Council NSW, Sydney, NSW Australia; 4 School of Exercise and Nutrition Sciences, Institute for Physical Activity and Nutrition (IPAN), Deakin University, Melbourne, Australia; 5 Physical Activity Laboratory, Baker Heart and Diabetes Institute, Melbourne, Australia; 6 Medical Oncology, St Vincent’s Hospital, Melbourne, Australia; 7 Melbourne, Australia; 8 Department of Physiotherapy, Faculty of Medicine Dentistry and Health Sciences, Melbourne School of Health Sciences, University of Melbourne, Melbourne, Australia; 9 Health Services Research and Implementation Science, Peter MacCallum Cancer Centre, Melbourne, Australia; 10 Sir Peter MacCallum Department of Oncology, The University of Melbourne, Melbourne, Australia; 11 Centre for Epidemiology and Biostatistics, Melbourne School of Population and Global Health, University of Melbourne, Melbourne, Australia

**Keywords:** Female, Breast neoplasm, Neoplasm, Exercise, Qualitative research

## Abstract

**Purpose:**

Physical activity can improve health in people living with and beyond breast cancer; however, how to best support physical activity participation in this population is unclear. This qualitative study sought to identify important physical activity program components for breast cancer.

**Methods:**

Women with previous breast cancer (*n* = 11) and allied health professionals (*n* = 7) participated in one-on-one semi-structured interviews (*n* = 15) or focus groups (*n* = 1). Qualitative data were analyzed using reflexive thematic analysis methods.

**Results:**

Four main themes were generated including (1) the need for physical activity programs; (2) person-centered programs; (3) flexible physical activity programs; and (4) systems factors. These reflected the health and non-health benefits of physical activity, the need to facilitate agency, the diversity in individual characteristics, preferences, abilities, and commitments of people with lived experience of cancer, as well as the need for physical activity programs to be integrated within the broader health system.

**Conclusion:**

Strategies to support physical activity engagement for breast cancer should embrace the diversity of those who are diagnosed with cancer as well as the diversity in which physical activity can be achieved.

**Supplementary Information:**

The online version contains supplementary material available at 10.1007/s00520-023-08113-7.

## Introduction

Breast cancer is the most common cancer among women worldwide with more than 2.2 million new cases diagnosed in 2020 [25]. While improvements in treatment and detection mean women are now surviving for longer after a breast cancer diagnosis, the impact of cancer and its treatment contribute to many having ongoing health care needs [15]. Symptoms including pain, fatigue, anxiety, and depression are frequently reported by women who have had breast cancer, which has a detrimental impact on quality of life [15]. Survivors also are at risk of cancer recurrence and have an increased mortality risk from subsequent primary tumors [26].

Physical activity can improve the quality of life of women living with and beyond breast cancer [2]. There is strong evidence that regular physical activity can decrease fatigue and depressive symptoms and emerging evidence indicates that it can reduce painful conditions like aromatase inhibitor-induced arthralgia [3, 8, 17]. Observational studies suggest that reducing sedentary time and performing more daily physical activity after diagnosis may reduce cancer recurrence and improve survival [8, 20, 27]. Given this, guidelines now recommend that physical activity interventions be integrated into routine cancer care [10, 24].

Despite these guidelines, few people with cancer achieve the minimum recommended physical activity levels [12, 16], have access to structured exercise programs [13], or have interest in available exercise interventions [1, 22]. Several factors underlie this. For example, most conventional cancer services, including exercise and rehabilitation programs, are limited to inpatient and outpatient settings and the availability of services declines with time after treatment [21]. Furthermore, in physical activity and cancer research, most studies have focussed on identifying benefit via tightly controlled efficacy trials. There has been much less research on dissemination and implementation [11], and therefore less is known about how to best design, deliver, and support physical activity programs for breast cancer. This qualitative study explored the components of physical activity programs that are considered important by women who have had breast cancer as well as the allied health staff that support them.

## Methodology

### Study design

This study utilized a qualitative design with reflexive thematic analysis [6, 7]. Reporting is in accordance with the consolidated criteria for reporting qualitative research (COREQ) [28].

### Participants and recruitment

Participants were recruited via convenience sampling, with advertisements placed on social media and distributed via the networks of support and advocacy organizations such as Cancer Council Victoria and Breast Cancer Network Australia. Eligible participants included adult (> 18 years) women who have had breast cancer who had completed their primary treatment at least 6 months prior, as well as allied health professionals who have spent at least a year working with people who have any cancer. Eligible participants were based in Australia and able to participate in a focus group or interview conducted in English. This study did not seek to achieve data saturation, defined as a point of information redundancy, as redundancy was not considered a need to satisfy the study purpose (i.e., idea generation) and as the principles of saturation are not consistent with those of reflexive thematic analysis [5, 9]. Rather, we estimated that a lower limit of 12 participants and an upper limit of 24 participants would be needed to provide rich and diverse perspectives regarding how to best support physical activity programs in women with breast cancer.

### Data collection

Participants could choose to attend either a single, hour-long focus group discussion or a single, 30-min, one-on-one semi-structured interview. Focus groups and interviews were scheduled at times convenient to participants, took place online, and were audio recorded. Investigators also took written notes during the focus groups and interviews. Focus groups and interviews were chaired by either investigator F. I. or C. S. F. I. is a female clinical trials manager who was conducting a Master of Cancer Sciences degree and who had experience working with people with cancer in research contexts. C. S. is a male exercise physiologist and physical activity for cancer researcher with prior experience in conducting qualitative interviews.

Participants with a cancer history were asked to give a brief outline of their cancer experience, while allied health professionals were asked about their work setting and their history of working with people who have cancer. Focus group and interview discussions then explored thoughts, experiences, and perspectives on physical activity participation and design elements of physical activity programs. The interview guide sought to identify what design elements and types of support are most important to women who have had breast cancer. That is, when provided with the scenario that new community-based physical activity programs for women with cancer were going to be developed, what factors would be important? The interview guide was informed by prior physical activity systems research, which recognizes that physical activity is a complex behavior [19], as well as clinical and lived experience perspectives of the investigator team. The interview guide and example questions are presented in Table 1.

**Table 1:** Example interview and focus group questions

Questions for allied health professionals	Questions for participants with lived experience of cancer
What do you consider when you prescribe exercise or physical activity to people who have had breast cancer? How do you consider symptoms like arthralgia or pain? Are there specific exercises you find useful? Or aggravating? Do you feel there are good programs for people who have had breast cancer to continue exercise after inpatient or outpatient rehabilitation? If so, what makes them good? And what are the limitations? If you were delivering a physical activity program for people who have had breast cancer and have ongoing symptoms, what kind of support would you want—for yourself? And for the patients? What social factors are important for patients? (e.g., patient groups) What environmental factors are important for patients? (e.g., safety) What technological factors could be beneficial to patients? (e.g., fit bits, telehealth)	Have you had any experience with an exercise or physical activity program as part of your cancer care? If yes, what was good about this service? Would you change anything? If no, what would a good program look like? Are there any physical activity services that you would like to use or avoid? (an example might be a gym) What are some of the personal things you would want someone to know if they were developing a new physical activity program for people who have had cancer? (e.g., exercise considerations for symptoms like pain) What type of information and support would be helpful from health care providers? (e.g., information on what type of exercise is best) Is it important for physical activity to be social? How could this be achieved? What are things about the physical environment, like where you live or work, or places you might exercise in, that could be important? (e.g., equipment or safety) Are you aware of technology that you would find useful or would recommend to others? Are there drawbacks to using technology for physical activity? (e.g., Fitbit, telehealth)

### Analysis

Participant descriptive data is presented using frequencies and percentages. Qualitative data were analyzed using a reflexive thematic analysis [6, 7]. This acknowledges the subjectivity of the researchers, which is used as a tool during analysis, and conceptualizes that themes are produced by the researchers through their engagement with the data. Two researchers (F. I., C. S.) completed data familiarization independently via re-listening, reading, and re-reading audio recordings and transcriptions. Initial codes were developed to help identify important features of the data relative to the physical activity program design. Initial candidate themes were generated by examining the codes and data were collated to identify patterns in participant responses. Candidate themes were then reviewed and refined via comparison to the collated data and via discussion between investigators F. I. and C. S. Theme names, descriptions, and corresponding quotes were then shared and reviewed by the broader team of study investigators. Supporting quotes presented have been edited for grammar and brevity.

## Results

### Participant characteristics

Between April and November 2022, 22 individuals responded to study recruitment materials. Of these, 18 individuals, including 11 participants with a history of cancer and seven allied health professionals, agreed to participate. Participants with a history of cancer included women living in either metropolitan (*n* = 9) or inner regional locations (*n* = 2). They reported treatment including surgery (*n* = 11; bilateral mastectomy = 4, lumpectomy = 4, “surgery” = 3), chemotherapy (*n* = 10), and/ or radiation therapy (*n* = 5). All participated via semi-structured interviews, which had a median (range) duration of 35 (28–67) min. Allied health professionals included exercise physiologists (*n* = 4), physiotherapists (*n* = 2), and nursing staff (*n* = 1). They worked in both hospital settings (*n* = 5) and community/private practice (*n* = 2). Allied health professionals had worked within cancer settings for a median (range) of 4 (1–11) years. Three allied health professionals participated in one focus group (duration = 40 min); the remainder participated in semi-structured interviews, which had a median (range) duration of 28 (13–49) min.

### Key themes

Four key themes were generated from the data. These are summarized in Table 2 and outlined below. An extended version of this table, with additional supporting quotes, is provided in Online Resource 1.

**Table 2:** An overview of themes and subthemes

Theme	Subtheme	Description	Example supporting quotes
1. The need for physical activity programs	Exercise as medicine	Participation in physical activity for health benefits	“I think from a side effects thing you feel so fatigued and, annoyingly, exercise helps with that. The joint pains that you get, I noticed that if I take a few days of not exercising, they’re more prominent, more inflamed maybe, and I guess as well like with the menopause aspect and that weakening of your bones there’s a greater importance of doing that resistance work, and that bone strengthening work.”—P2
	Beyond exercise as medicine	Participation in physical activity for reasons unrelated to specific health benefits	“I’ve found walking when I was going through cancer and everything I used to just walk down to the beach and just watch the sunset. It was only a short walk for me but focusing on what was beautiful about the world, it’s really important.”—P6
	Continued care	Need for programs outside of hospitals to support continued activity	“Where do you find clubs for women over 50 to be active”—P9
2. Person-centered programs	Individualized prescription	Allied health professionals consider who the participant is and what they want when prescribing physical activity	“Prescribing exercise in a holistic way. So not focusing on just the issue, focusing broadly on the person.”—AH1
	Participant agency and autonomy	Individuals can make informed decisions about their own physical activity	“I don’t want to be treated like someone who isn’t like a full participant in it. I’d rather have agency.”—P3
3. Flexible physical activity programs	Flexibility in who	Variation in age, fitness, interests, commitments, culture, and cancer experiences of people with lived experience	“It’s a square peg in a round hole. Yeah, they’re saying I have to fit the program. Not the program to fit me.”—P5
	Flexibility in what	People are interested in different types of physical activities for different reasons	“It’s very important to have different kinds of exercise, different types. What I see in myself and others is like I think exercise is so bad, so I don’t go, I don’t want to go because I don’t like the gym. Thinking that exercise is only one possibility, I think we have to have other options”—P10
	Flexibility in mode and setting	No single mode or setting will suit all people at all times	“As part of life with cancer, there’s so many appointments and you’re just like Oh my God, it’s like relentless and so then being able to not leave your house and do it would be good.”—P1
4. Systems factors	Funding and program ownership	Physical activity as a business implies cost which creates barriers and can affect equity	“They were scrambling for funding and I, to be honest, I thought, yep, here we go again for women’s health. You get something that works and then the next thing you know it’s stopped.”—P6
	Connection to other health care services	Physical activity programs cannot exist in silos and are more likely to be successful when they are supported by the greater system	“There is definitely a gap, as far as I can tell, between the immediate oncology care, whether that’s inpatient or outpatient, and then the connection to other services. That’s certainly been a barrier that I found to trying to develop a community private practice service that’s oncology focussed – to actually have that consistent referraling.”—AH5

Key: *AH* allied health participant, *P* participant with lived experience of cancer

### The need for physical activity programs

Overall, participant responses validated the need for physical activity programs, describing a range of benefits and reasons for participating. The first subtheme, exercise as medicine, reflects that people may elect to participate in physical activity as an adjunct therapy for cancer care. Participants outlined challenges experienced after cancer including loss of muscle and physical function, as well as fatigue, pain, and stiffness. They perceived that being physically active could help address these impacts of cancer and potentially reduce breast cancer recurrence.

My reasons are to reduce my risk of getting cancer, but also not to exacerbate my stiffness and the pain that I do get. I feel that doing my exercise means that I'm not going to make that worse. I'm going to make you know, kind of keep it at a reasonable tolerable level.—Lived experience participant (P) 5

Beyond exercise as medicine reflects that the reasons for being physically active extended beyond potential therapeutic benefits. Participants described a desire for something that can be fun and distracting and could restore self-worth and provide structure after treatment, and something that could facilitate engagement in other areas of life.

So, the rehab program really helped because it gave me routine. I had routine with the hospital and then I had nothing for three months. And then it was I got something to grasp onto something.—P4

Having community-based programs facilitates continued care. Allied health staff working in hospitals commented on the absence of good cancer-specific programs available in the community that people could engage with after discharge. This can limit the overall benefit achieved in inpatient and outpatient settings.

I feel that because we don’t have the appropriate or, I suppose more funded, public health approaches in the community that transition or those beneficial effects that we’ve sort of provided and educated can sometimes be left astray as soon as they leave our service.—Allied health (AH)2 (exercise physiologist)

### Person-centered programs

The second theme addressed the role of participants in physical activity programs. All allied health professionals considered individual needs, abilities, and goals when prescribing physical activity, suggesting that a level of patient-centered care is already embodied within allied health practice. For some, developing independence and an understanding of how to engage in physical activity was a primary goal of rehabilitation and they actively looked to build the capabilities and understanding required.

What I say to my patients is that what I really want for you in physiotherapy is to build exercise literacy. I want you to learn how to pace your own exercise. How to do a little bit more, how to do a little bit less, and how does it feel to do that ongoing with cancer—AH7 (physiotherapist)

The subtheme of agency and autonomy reflects that people with lived experience do not simply want to be passive participants in physical activity programs. This was evident when reflecting on the level that lifestyle for cancer materials should be presented. That is, with sufficient detail to facilitate autonomy and informed choices. This was also reflected when discussing models of support. For several participants, supported self-management, where individuals take responsibility for their own physical activity but can check in periodically with an exercise or health professional, was desired.

What I need 12-months on is to be able to go back and say, see this is what I’m doing, this is how I’m doing it. These are my current physical kind of limitations or pain and stiffness concerns. Can you let me know do I need to increase weights, reduce weight, change some of my exercises and that could just be once every three months—P5

People with lived experience can also deliver physical activity to others. One participant, after having participated in an exercise for cancer trial, was retraining as a personal trainer with the goal of working with and advocating for physical activity as a part of cancer recovery. For some with cancer, physical activity that is delivered by someone with lived experience may have additional benefit.

(Our trainer) also had a double mastectomy, so she had been through what a lot of us had been through, and in some cases more than what we’ve been through. So, the empathy was there straight away. And she knew about how embarrassed you might feel about taking your clothes off in the changing rooms.—P6

### Flexible physical activity programs

Physical activity programs need to be flexible to cater to diverse needs and preferences. Participants commented that their age, fitness, work or family commitments, and cultural background meant that existing physical activity for cancer programs often did not suit them. Some participants felt the programs they tried were too gentle. Work or family commitments prevented people from attending programs offered only during the day. Furthermore, common terminology, such as “survivor,” excludes people living with cancer, particularly metastatic cancer, who still need physical activity support even though they have not wholly completed treatment.

To appeal to a large range of people, programs need to be flexible in the type of physical activity they offer. Participants described a range of different activities that they were and were not interested in, as well as a range of reasons they enjoyed them. Activities mentioned included running, swimming or aquatic-based exercises, paddle boarding, arts-based activities like dance or sculpture building, and skill-based activities like archery or shooting.

I’m kind of getting back to that (pre-treatment) pace some in some ways. I’m a little bit better and I’m training for a half marathon in February.—P2

The activities I’m looking for are things where I don’t run around and get out of breath and are maybe skill based. That is essentially the criteria for me.—P7

The ideal delivery mode and setting will vary with treatment and recovery stage, as well as personal preference. Gyms were viewed with some caution, especially when impacts of the cancer were still visible. Online programs were discussed positively when participants were unwell, although could become boring. Social programs may be good for many, but not be assumed all people with cancer want this. Physical activities delivered in outdoor areas and community settings were also considered appealing in that they offer a contrast to hospital waiting rooms or doctors’ offices.

All these patients are inside a hospital or inside a waiting room all the time, constantly, during treatment, getting out of treatment, or waiting for a doctor. So, something that’s an outdoor activity or venue or something that’s away from the hospital maybe or something that’s a bit nicer than constantly being on the hospital grounds.—AH6 (nurse)

### System factors

Physical activity for cancer programs cannot exist in a silo. Programs are dependent on funding, referrals, and relationships with other health care organizations. Funding and program ownership was a subtheme that emphasized that although people desire equitable services, programs cost money. Allied health professionals acknowledged this as something that affects service provision, referrals, and the reality of running a business.

Unfortunately, we’ve got to charge for our services. I’ve got rent to pay and staff to pay and at some point, myself to pay—AH5 (exercise physiologist)

Physical activity programs also need to be connected to other health services and cancer organizations to be sustainable. This increases visibility and referrals and may protect community-based professionals against a feeling of isolation. Having said this, while allied health professionals in hospitals do want community services to refer patients to after treatment, becoming and remaining connected to these services is not simple and is instead time intensive.

No (we don’t have good options to refer after discharge). Especially being a specialist Cancer Centre so people aren’t within our post code, it’s really tricky because they’re coming from all over the state and country. So, for clinicians to try and understand local services Australia wide is really challenging.—AH3 (physiotherapist)

Alternative referral pathways and models of ownership were discussed. General practitioners and cancer organizations were identified as potential referral partners, with experiences obtaining referrals via cancer organizations described as more positive and consistent than via general practitioners. Cancer organizations, along with health funds and councils, were identified as potential program owners or funders, which could help sustainability.

## Discussion

This study sought to generate knowledge and ideas pertaining to the development and support of physical activity programs for people living with and beyond breast cancer. While participants validated the need for physical activity programs, responses clearly indicated that a physical activity program for cancer is a complex intervention, with diversity in why, how, what, when, and where people will engage. No single physical activity program will be able to cater to all people with breast cancer, and a range of diverse and flexible approaches are needed.

This study has several key strengths that underlie its ability to provide novel qualitative findings on how to best deliver and support physical activity in women who have had breast cancer. Participants described a wide range of considerations for physical activity program developments and were able to provide practice-based perspectives. The study also has some limitations. While participants were invited to describe their cancer experiences or their experience in allied health, the study did not collect specific demographic or baseline physical activity data and is therefore limited in its ability to extensively describe participants. The study did not seek to achieve data saturation and does not attempt to represent the views of all women who have had breast cancer or all allied health professionals. It is likely that those who participated already had some interest in physical activity and their responses may not reflect the preferences of those who are inactive. Furthermore, most allied health staff who agreed to participate were exercise professionals (e.g., physiotherapists, exercise physiologists). While this is a strength, as these individuals can offer unique practice-based perspectives regarding physical activity delivery, a broader group (e.g., psychologists, social workers, yoga instructors, personal trainers) may have provided additional perspectives on support features not otherwise captured. Hospital-based allied health staff described the limited availability of community-based programs for people with cancer, but this does not imply there are no suitable programs. Rather, it may reflect their awareness of the different options.

Several themes from this study are consistent with those generated by other research into physical activity for cancer. For example, in the current study, participants validated the need for physical activity programs, which agrees with quantitative evidence for positive health effects of physical activity [8, 24], qualitative studies where people with cancer describe their desire for, and benefits from, physical activity [4, 14], and health service surveys that have identified relative sparsity of programs outside of hospitals [13, 21]. Participant comments that existing physical activity programs for cancer often do not align with their physical interests or capabilities or did not have the flexibility that allowed them to attend with concurrent work or family commitments, which is consistent with common reasons for declining to participate in exercise for cancer trials [22]. Furthermore, the importance of factors beyond the physical activity program, such as funding and the need for support from other components of the health care system, has previously been identified as barriers to implementation in exercise oncology [18].

This study also contained themes that were distinct from previous findings. For example, while previous studies have described walking as the most preferred mode of physical activity across cancer types and the favorite activity type of all women with breast cancer [14, 23], in this study, participants described a wide range of physical activities that they would and would not enjoy. The active role of participants in physical activity programs is also distinct from themes outlined in some prior studies that have highlighted a preference for exercise for cancer that is supervised by experts [4, 14]. In this study, participants emphasized their agency, their desire to make their own informed decisions, and their potential role in leading physical activity for themselves or others. Importantly, this theme is not in disagreement with the value of expert support and does not imply that there is no need for qualified exercise professionals. Rather, it highlights that there are multiple ways of delivering physical activity programs that may be acceptable to people with cancer.

This study has several implications for the design and delivery of physical activity programs for people with cancer. While several participants exercised to achieve health benefits, others participated in physical activity for fun, for structure, or to engage with nature. As such, while creating and marketing physical activity programs for health benefits is important, creating alternate interventions that still support physical activity without being about physical activity per se, such as gardening, dance, or other arts-based programs could reach a broader group. Similarly, the range of activities participants were interested in also has implications for how to support physical activity in breast cancer. Strategies that promote a single activity type (e.g., walking groups or walking maps) or a single delivery method (e.g., telehealth) or rely on a specific setting (e.g., a gym) may appeal to some people living with or beyond cancer, but initiatives that emphasize the many different types of physical activity will have broader reach.

Both participants with lived experience and allied health professionals indicated that the success of a new physical activity program for people with cancer is not just dependent on developing the right content for the population and setting, but also on factors like the ability to obtain funding or the ability to build relationships with other sectors of the health care workforce that can provide referrals. Neither are simple and should not be dependent on individual clinicians or community providers. Rather, the government, peak bodies, and advocacy organizations could help facilitate this.

## Conclusion

The themes from this study emphasize the considerable diversity and flexibility required when designing, constructing, and delivering physical activity interventions for women living with and beyond breast cancer. However, program development ideally follows an iterative process, and future research should employ a multi-stage, co-design process to develop sample programs that can then be appraised and then delivered to the community.

### Supplementary Information

Below is the link to the electronic supplementary material.

Supplementary file1 (DOCX 23 KB)

## Data Availability

De-identified data may be made available upon reasonable request.
