# Supplementary material for: Diverse strategies are needed to support physical activity engagement in women who have had breast cancer
Source: Support Care Cancer. 2023 Oct 21;31(11):648. doi: 10.1007/s00520-023-08113-7 (PMC10590305; doi:10.1007/s00520-023-08113-7)
Supplement: Supplementary file 1 — Supplementary file1 (DOCX 23 KB) [file 520_2023_8113_MOESM1_ESM.docx]

**Diverse strategies are needed to support physical activity engagement in women who have had breast cancer.**

Farha Inam, Rebecca Bergin, David Mizrahi, David W Dunstan, Melissa Moore, Natalie Maxwell-Davis, Linda Denehy, Brigid M Lynch, Christopher TV Swain

**Online Resource 1: Study themes and subthemes with extended descriptions and supporting quotes**

| **Theme** | **Subtheme** | **Description** | **Example supporting quotes** |
| --- | --- | --- | --- |
| 1. The need for physical activity programs | Exercise as Medicine | Participants identified need for physical activity after cancer and its treatments | “I lost a lot of my muscles with chemo … And so it's hard to bath the baby, get the groceries in and out of the car. You know, that kind of stuff.” – P3 |
|  |  | Participants maintained physical activity because of specific exercise as medicine benefits | “One of the biggest draw cards for people with cancer to exercises that exercise reduces the chance of recurrence. ... You know chemo makes you basically a shell of yourself and it's hard, so you want to do anything and everything in your power never to be in that space again." - P4 |
|  | Beyond exercise as medicine | Participation in physical activity for reasons unrelated to specific health benefits. | “I've found walking when I was going through cancer and everything I used to just walk down to the beach and just watch the sunset. It was only a short walk for me but focusing on what was beautiful about the world, it's really important.” – P6  “I am a health professional, but I didn't think in this way. I just think that I was feeling good doing something during this time because it's a hard time.” – P10 |
|  | Continued care | Participants commented on the absence and impact of programs available after treatment. | “There wasn't much focus on fitness. Obviously after a bilateral mastectomy, you do get some physio exercises to do, but there wasn't a heap of follow up or anything like that. I think in general, there's not enough focus on exercise as part of recovery. ... I didn't see anything. Nobody spoke to me about it and I didn't really see it.” - P1 |
| 2. Person centred programs | Individualised prescription | Prescribing exercise suitable to the individual was discussed more than encouraging participants to meet guidelines. | “I think it comes down to individualization, and I suppose having a very good understanding of the case and the patients experience.” – AH2 (Exercise physiologist) |
|  | Agency and autonomy | Individuals can make informed decisions about their own physical activity. | “To be told to eat healthy and exercise every day is maybe level one and it’s great to be told that once, maybe twice, but then next time you want to know about what exercise I should be doing or what it’s based on. You know, what are the recommendations are based on? Was there any research? What was the research? And sometimes that is a bit hard to track down.” – P5 |
|  |  | People also have an interest in delivering physical activity programs to others who have had cancer. | “Exercise played a really big part in my treatment last year and it's become a big focus of mine now. So much so that I'm actually looking into training to be a personal trainer to work with cancer patients.” – P2  “I would start one (a program) today if I had the money” - P9 |
| 3. Flexible physical activity programs | Flexibility in who | Variation in age, fitness, and goals mean that existing physical activity programs were unsuitable. | “Some of the frustration I had with a lot of the free services was they were targeted to an older demographic, and I felt that they were a little bit too gentle. Yeah, maybe too gentle for where I was at or where I wanted to be.” – P2  “I was already, probably, you know a good 12 months after the end of my treatment, and it had a lot to do with focusing on people who hadn't even started the journey of exercise.” – P5 |
|  |  | Work and family commitments are a barrier to attending programs only offered at set times or in set facilities. | “There are the logistics for me. Like childcare. … Any exercise class or equipment with young kids was tricky.” – P8  “You join up for a group program and the group program happens during working hours. So, it wasn't going to work for me” – P5  “A lot of my exercise was sort of delayed because I was a single mum. I had a daughter that just started uni and one that was just finishing and I was working part time teaching. I mean, I went back to work while I was going through chemotherapy. I could not afford not to work.” - P6 |
|  |  | Terminology, like survivor, can limit who can attend. | “Every (survivorship) program you try and get services for they always ask you ‘When have you finished treatment?’ and if you say no, then they'll say, ‘come back when you have.’ … I try and raise the flag on metastatic cancer. You know people who are living quite well and who need support and need exercise to help them stay healthy.” – P4 |
|  |  | Desire for programs that are culturally sensitive. | “I would like a trainer who understands my culture and (empathises) with my cancer experience” - P11 |
|  | Flexibility in what | People are interested in different types of physical activities for different reasons. | “Swimming to me is very important. Just doing exercises in the water after you’ve had quite a traumatic experience. It is like there are healing elements of water.” - P6  “I’d like different activities that can distract, like paddle boarding” – P9  “I don’t think any one intervention is going to get a significant majority of people. Particularly with cancer you have a group of people with really mixed backgrounds. You need to offer a variety of programs.” – AH4 (Exercise physiologist) |
|  | Flexibility in mode and setting | There was caution regarding gym-based settings. | “I found it a little bit difficult reintegrating to normal gym spaces where everyone else is, on the outside, normal, and healthy. This was particularly true when I had shorter hair that was growing back from chemo or if you can see your scars. That was probably a little bit difficult.’ – P2  “The thing that perhaps stops them from going to a regular gym is the horror stories of personal trainers asking you to drop and do 50 push-ups, right? Like we don't have that ability.” - P4 |
|  |  | Online and remote programs were good when people felt unwell, but questions about whether these could sustain interest. | “I think when I went through treatment it was during COVID, so a lot of my stuff was virtual and in some respects it was good because throughout treatment, particularly chemo, you're very wiped out and low on energy and so being able to kind of do that in the comfort of your home was nice and with just one person as well, it felt a bit more comfortable.” – P2  “With the pandemic I started yoga online. I loved it, but not for a long time because it's boring online … Online will be a good option for people who are not able to see others because of the treatment, their body, the symptoms. But I am 90% certain that after this moment the majority of the people will want to go outside” - P10 |
|  |  | Social programs may encourage a sense of belonging and continued exercise, but not everyone wants this. | “Like peer support, so other people going through it so then you can create a little group and then those people could support each other as well. … it would kind of bleed into other conversations about food and all that all that other stuff.” – P1  “I had an experience at my previous workplace, which was hospital based, where we had a really lovely group of people that established a really lovely friendship and they actually continued to do outdoor exercise together as a group ongoing for 12-months post discharge.” – AH1 (Exercise physiologist)    “It was ‘If you come for the exercise sessions, you're also in the program to go to the peer support session’, and it's that kind of well and good but I don't want those parts, I just want the parts that I need.” – P5  “I think I'd rather do things by myself. And whether that means doing it at home or outside by myself, it doesn't really matter.” – P7 |
|  |  | Appointments during cancer and time in hospital can be overwhelming. Settings that are distinct to this may be of value. | “I was in Olivia Newton John Center and that's just amazing. You go in and there’s a piano in the waiting room. The waiting room has couches and magazines and it's not like chairs along the hallway. It's really nice and it doesn't feel very hospital-y or medical at all.” - P1  “Where possible it would be good to use an outdoor area. On the coast we have so many great beaches, bushlands, and stuff like that. And all these patients are inside a hospital or inside a waiting room all the time, constantly, during treatment, getting out of treatment, or waiting for a doctor. So, something that’s an outdoor activity or venue or something that’s away from the hospital maybe or something that’s a bit nicer than constantly being on the hospital grounds.” - AH6 (Nurse) |
| 4. Systems factors | Funding and program ownership | The cost of physical activity programs can limit accessibility and sustainability. | “If you’re going to create something … You've got to address equity … I think it's really important that with any illness, if you find a way of organising a program that people can get access to, that's going to help them, that it's offered out there.” – P6  “If patients can afford it, they are probably OK. But if money’s an issue, then it becomes really challenging.” – AH3 (Physiotherapist) |
|  |  | Alternative models of ownership, like through councils, health funds, and support organisations may help. | “There's a way of doing it because again health funds want to keep their people healthy, they don't want them to get cancer again ... because cancer patients cost them a lot of money, and if there's preventative programs they would be, I'm sure, quite open to covering it.” - P4 |
|  | Connection to other healthcare services | Physical activity programs cannot exist in silos and are more likely to be successful when they are supported by the greater system. | “You need to have open communication lines between all of the practitioners involved and you need all of those practitioners to be interested in that communication. You know it's very easy for me to send letters to surgeons, but whether they actually pay attention and read through those letters, it is a different thing.” – AH4 (Exercise physiologist). |
|  |  | Referrals can come from GPs and cancer charities, but these pathways can be mixed. | “I do remember I asked for an enhanced primary care program with my GP. I asked for a referral to an exercise physiologist and physiotherapist. And I think he just looked at me and said you're a 60-year-old woman. You should do this this, this, and this, see you later. And I just felt that he didn't really look at me as a person and didn't ask me what I could do. What I normally did.” – P5  “The two referral partners that have jumped on board the most have both been oncology nurses. One is a prostate foundation nurse, and one is a McGrath foundation nurse but if it wasn’t for those people we would have real difficulty.” - AH5 (Exercise physiologist) |

Key: AH = Allied health participant; P = participant with lived experience of cancer.
